# Epitranscriptomic Analysis of A-to-I RNA Editing and m^6^A Using Short- and Long-Read Sequencing Technologies

**DOI:** 10.3390/ijms27135858

**Published:** 2026-06-29

**Authors:** Nicholas Brenna, Domenico Alessandro Silvestris, Silvana Zugaro, Elena Orecchini, Enrica Crivaro, Laura Leo, Angela Gallo

**Affiliations:** 1 Department of Onco-Haematology, Genetics and Epigenetics of Pediatric Tumours, Bambino Gesù Children’s Hospital IRCCS, 00146 Rome, Italy; nicholas.brenna@opbg.net (N.B.); silvana.zugaro@opbg.net (S.Z.); elena.orecchini@opbg.net (E.O.); enrica.crivaro@opbg.net (E.C.); laura.leo@opbg.net (L.L.); 2 Department of Biosciences, Biotechnology and Environment, University of Bari Aldo Moro, 70125 Bari, Italy; 3 Department for the Promotion of Human Science and Quality of Life, San Raffaele University of Rome, 00166 Rome, Italy

**Keywords:** epitranscriptomics, RNA modifications, m^6^A, RNA editing, long-read sequencing, direct RNA sequencing, Illumina

## Abstract

More than 160 types of post-transcriptional RNA modifications have been identified, revealing considerable diversity in their types, abundances, distributions, and functional roles across different RNAs, cells, and tissues in humans. Recent advances in high-throughput sequencing technologies have enabled the systematic detection of dynamic RNA modifications, including N6-methyladenosine (m^6^A) and inosine (I). In this review, we focus on RNA modifications in eukaryotic mRNA and provide an overview of current high-throughput methodologies for detecting the most abundant adenosine-related modifications, including m^6^A and adenosine-to-inosine (A-to-I) RNA editing. Finally, we discuss the major challenges that remain in the field and highlight key directions for future research.

## 1. Introduction

**Figure 1:**
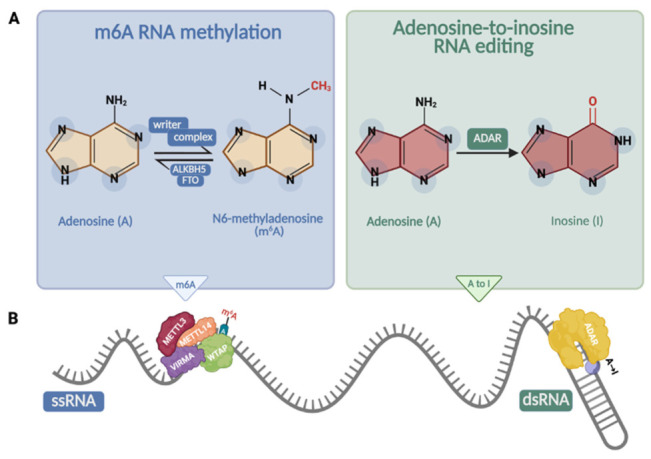
(**A**) Overview of two major epitranscriptomic modifications: m^6^A and A-to-I RNA editing. m^6^A is deposited on single-stranded RNA by the METTL3-METTL14 writer complex and removed by demethylases (FTO, ALKBH5), whereas A-to-I editing is catalyzed by ADAR enzymes on double-stranded RNA, converting adenosine to inosine. (**B**) Structural context of these modifications: m^6^A occurs predominantly on ssRNA, while A-to-I editing targets dsRNA regions, reflecting distinct regulatory mechanisms. Created in BioRender. Gallo, A. (2026) https://BioRender.com/hncgszr, accessed on 25 March 2026.

RNA molecules undergo a variety of chemical modifications that enhance their functional potential within the cell. These epitranscriptomic modifications constitute a critical layer of post-transcriptional regulation, influencing RNA stability, localization, structure, and interactions with proteins and other RNAs, thereby shaping gene expression programs in both physiological and pathological contexts [1]. RNA modifications are introduced co- or post-transcriptionally and can be reversible (e.g., m^6^A), enabling dynamic regulation of gene expression without altering the underlying genetic sequence [2,3,4,5]. In contrast, other RNA processing events like adenosine-to-inosine (A-to-I) editing can alter nucleotide identity, resulting in recoding effects that have significant functional implications [6]. Among the more than 160 RNA modifications described to date [7], only a subset is present in eukaryotes/mammals; while no exact number is defined, this subset clearly exceeds ~50 modifications, given that tRNA alone contains more than 50 distinct modifications [8]. Of note, several core RNA modifications are conserved from bacteria to humans, including pseudouridine, m^6^A and inosine (primarily in tRNA wobble positions) [8,9]. This review focuses on two of the most extensively studied and biologically relevant adenosine-associated modifications: A-to-I and N6-methyladenosine (m^6^A). These modifications provide complementary models for understanding both the biochemical diversity and the computational challenges associated with epitranscriptomic events (Figure 1).

A-to-I RNA editing is catalyzed by the Adenosine DeAminases acting on dsRNA (ADAR) family of enzymes including the two active deaminases ADAR1 and ADAR2 and the catalytically inactive ADAR3 [10]. Double-stranded RNA structures are common in humans, as they mostly arise from inverted repeat elements such as Alu sequences [11]. A-to-I editing occurs widely in pre-mRNAs, mRNAs, and long non-coding RNAs (lncRNAs) but also within non-coding RNAs such as microRNAs (miRNAs) precursors [12,13,14,15,16]. The deamination of adenosine to inosine alters base-pairing properties, as inosine preferentially pairs with cytosine rather than uridine. This preference arises because inosine lacks the exocyclic amino group of adenosine and instead features hydrogen-bond acceptors that form hydrogen bonds with cytosine, similar to guanosine [17]. Inosine is read as guanosine by the translation machinery and during reverse transcription (RT), leading to the incorporation of cytosine in the complementary cDNA strand. This principle allows its detection by Sanger sequencing prior to the advent of next-generation sequencing (NGS). As inosine behaves as guanosine during splicing and translation this conversion can lead to various outcomes, including transcript recoding, changes in miRNA targeting, and modulation of alternative splicing [18,19]. A recent RNA-seq study across various human tissues found 1741 A-to-I editing sites in coding sequences [20]. Such editing within CDS can lead to the inclusion of different amino acids in proteins beyond what is encoded by the DNA, which can have important consequences for health and disease [21]. Together with coding RNA, inosine has been found in non-coding RNAs, including miRNAs, lncRNAs, circular RNAs (circRNAs) [22] and tRNA, even if the deamination within tRNA results from enzymes other than ADARs [23]. A-to-I RNA editing events can also be detected by deep sequencing. Because inosine preferentially base pairs with cytosine rather than uracil, these editing events are detected as A-to-G mismatches when comparing genomic DNA and corresponding cDNA sequences. Such “mutational” RT signatures represent a straightforward strategy for identifying inosine residues in RNA. However, A-to-I editing can be incomplete and may be confused with sequencing errors or single nucleotide polymorphisms (SNPs). Despite this limitation and the relatively high false-positive rate, detection of inosine-associated mismatches remains a widely used approach for global profiling of the A-to-I editome.

m^6^A is a mark that deposits a methyl group on adenosine residues at the N6 position, catalyzed by the m^6^A methyltransferase complex (METTL3–METTL14 core plus regulatory subunits, such as WTAP, VIRMA, and RBM15). Unlike inosine generation, this mark is reversible and can be removed by the demethylases FTO and ALKBH5 [24]. Functional effects of m^6^A are mediated by “reader” proteins, such as members of the YTH domain-containing proteins, which recognize methylated adenosines and modulate all the steps of RNA metabolism involving alternative splicing, nuclear export, translation, and degradation [25,26]. Unlike A-to-I editing, m^6^A does not alter nucleotide identity or base-pairing properties; instead, it serves as a reversible chemical mark that influences RNA behavior through protein-mediated recognition.

This fundamental difference significantly impacts detection: while A-to-I editing can be identified through sequence mismatches, m^6^A does not produce a typical cDNA signature, making NGS alone insufficient for identification without supplementary methods [24]. Of note, m^6^A deposition preferentially occurs within a conserved DRACH motif (D = A/G/U, R = A/G, H = A/C/U). However, only a subset of DRACH motifs is methylated *in vivo* [24], indicating that sequence context alone is insufficient to determine methylation specificity. However, it has been reported that m^6^A is preferentially enriched within long internal exons and around stop codons, as well as in 3′ untranslated regions (3′ UTRs). Alongside mRNA, m^6^A has also been found in most non-coding RNAs, including ribosomal RNAs (rRNAs), small nuclear RNAs (snRNAs), small nucleolar RNAs (snoRNAs), miRNAs, lncRNAs, and circRNAs [27,28,29]. Following the major discoveries of extensive m^6^A methylation in mRNAs, numerous deep-sequencing methods and protocols have been developed. Many existing techniques rely on characteristic RT signatures induced by RNA modifications during cDNA synthesis; these signatures can be either naturally occurring or introduced through specific enzymatic or chemical treatments. Specifically, RT-based signature–inducing strategies can be achieved using synthetic base-modified dNTPs. Selenium-substituted thymidine (4Se-dTTP) that perturbs RT in the presence of m^6^A, generating characteristic RT signatures that can be used for its indirect detection [30]. Additionally, deep-sequencing approaches based on antibody enrichment (e.g., MeRIP-seq) can be used for m^6^A mapping at varying resolution.

**Figure 2:**
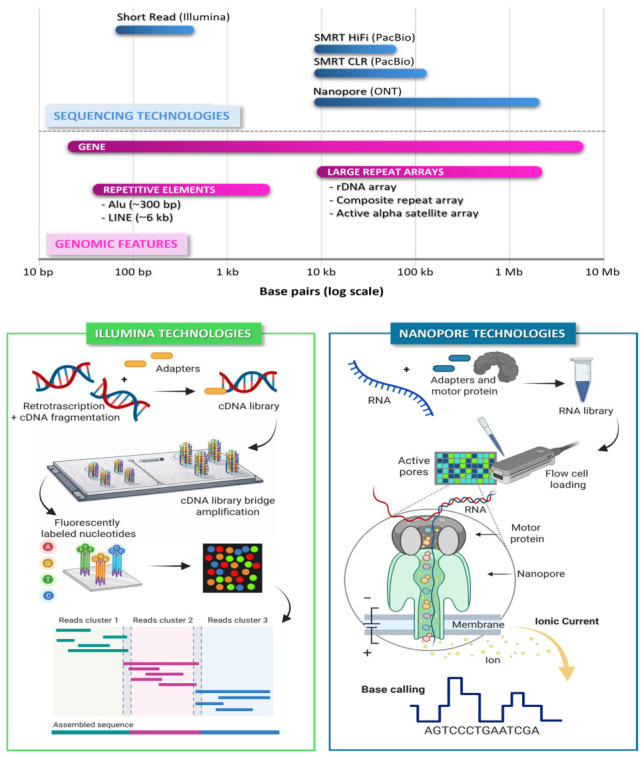
Comparison of short-read and long-read sequencing technologies. Schematic overview of sequencing paradigms and their characteristics. Short-read sequencing (e.g., Illumina sequencing-by-synthesis) generates fragmented cDNA reads, providing high accuracy and throughput but limited resolution of full-length transcripts and repetitive regions. In contrast, long-read technologies, including PacBio SMRT sequencing and Oxford Nanopore sequencing, produce substantially longer reads that can span entire transcripts and complex genomic regions. The lower panels illustrate the underlying principles of Illumina sequencing, based on bridge amplification and cyclic reversible termination, and Nanopore sequencing, which measures changes in ionic current as nucleic acids pass through a nanopore. While short-read approaches enable robust population-level analyses, long-read sequencing allows isoform-resolved and single-molecule investigation of transcript structure and modifications. Created in BioRender. Gallo, A. (2026) https://BioRender.com/14s82xn, accessed on 8 April 2026.

At present, two major technological paradigms dominate the field of m^6^A and A-to-I marks (Figure 2): (i) short-read sequencing-by-synthesis (SBS), primarily on Illumina platforms, which provides an averaged representation of RNA modification signals across populations of RNA molecules, and (ii) single-molecule long-read sequencing approaches (third-generation sequencing), which enable analysis of RNA molecules at the single-molecule level. Single-molecule sequencing technologies initially emerged with Pacific Biosciences (PacBio) single-molecule real-time (SMRT) sequencing, which facilitated long-read DNA sequencing and transcript isoform analysis using cDNA workflows (Iso-Seq). Later, Oxford Nanopore Technologies introduced nanopore-based sequencing, enabling direct sequencing of native RNA molecules (direct RNA sequencing, DRS). This approach enables the detection of RNA modifications by identifying distinctive signal deviations [31,32,33]. Illumina SBS relies on cDNA sequencing and offers high accuracy and throughput but does not preserve native RNA modifications and is limited by short read lengths. In contrast, Nanopore DRS sequences native RNA molecules directly, preserving chemical modifications, albeit with higher error rates and increased computational complexity.

The advancement of long-read sequencing techniques has significantly increased read lengths to several kilobases, thereby improving accuracy in repetitive regions. This is especially important for A-to-I editing events, as these modifications occur in repetitive elements like *Alu* sequences. Because long reads can span these regions and their unique flanking sequences, they reduce multi-mapping issues and enhance the precision of editing detection in repeat-rich regions. Comparing Illumina and Nanopore technologies is thus more than just a technical contrast; it reflects different insights into the epitranscriptome: Illumina methods deliver reliable, population-wide measurements of modification signals, while Nanopore sequencing allows examination of epitranscriptomic diversity at the level of single RNA molecules and isoforms.

This review outlines the evolution of sequencing technologies and the strategies commonly employed in contemporary epitranscriptomic analyses, with particular emphasis on poly(A) RNAs, m^6^A, and inosine. It also explores how different methodologies can influence diverse areas of biological research.

## 2. Decoding the Different Layers of the Epitranscriptome

Epitranscriptomic marks can be decoded through diverse strategies that uncover different kinds of biological insights: (i) at single-nucleotide resolution and (ii) by analyzing full-length RNA molecules and transcript isoforms, allowing the examination of individual or combined modification patterns within transcripts [25].

### 2.1. Single-Nucleotide Resolution

Transcriptome-wide identification of m^6^A using Illumina sequencing platforms is mainly based on antibody-dependent enrichment strategies followed by next-generation sequencing. The most widely used approach is methylated RNA immunoprecipitation sequencing (MeRIP-seq or m^6^A-seq), in which fragmented RNA is immunoprecipitated using anti-m^6^A antibodies and sequenced to identify regions enriched for methylation relative to input controls [34,35]. This method enables transcriptome-wide mapping of m^6^A peaks but does not provide single-nucleotide resolution. To achieve higher resolution, miCLIP (m^6^A individual-nucleotide resolution crosslinking and immunoprecipitation) exploits UV crosslinking-induced antibody–RNA interactions, generating characteristic mutations or truncations during RT that allow identification of m^6^A sites at single-nucleotide resolution [36]. Related CLIP-based strategies, including improved crosslinking and library preparation approaches, further enhance mapping accuracy and signal specificity [37]. In addition, quantitative approaches such as m^6^A-LAIC-seq allow estimation of the methylation fraction per transcript, providing information on modification stoichiometry rather than only positional enrichment [38]. However, all antibody-based methods are inherently limited by dependence on immunoprecipitation efficiency, antibody specificity, and the inability to directly provide absolute quantification of m^6^A levels. Recently, GLORI, a sequencing-based method combined with a dedicated bioinformatics pipeline (GLORI-tools), has been developed for transcriptome-wide m^6^A profiling at single-nucleotide resolution. This approach enables quantitative mapping of m^6^A without the use of antibodies, providing improved estimation of methylation levels compared to traditional immunoprecipitation-based methods [39,40]. GLORI uses glyoxal- and nitrite-mediated deamination of unmethylated adenosines while preserving m^6^A, enabling detection of methylation through sequencing-derived signatures. The resulting libraries are compatible with standard Illumina sequencing workflows. This method provides a highly sensitive and robust quantitative readout of m^6^A sites at single-nucleotide resolution and enables global estimation of methylation stoichiometry across the transcriptome. At single-nucleotide resolution, inosine detection remains technically challenging due to its chemical similarity to guanosine during RT. Methods such as ICE-seq [41] exploit selective chemical modification of inosine to induce RT stops or misincorporation signatures, enabling precise site mapping. More recently, enzyme-assisted approaches based on Endonuclease V (EndoV), which specifically recognizes inosine-containing RNA, have been adapted to achieve higher-resolution detection when coupled with high-throughput sequencing [42,43]. However, these strategies often suffer from incomplete specificity or efficiency, and their performance can be influenced by local RNA structure and sequence context. Additionally, direct detection of A-to-I editing at single-nucleotide resolution still relies on the identification of A-to-G mismatches in RNA-seq data, which necessitates rigorous filtering and integration with genomic DNA controls to distinguish true editing events from technical artifacts [44]. As a result, combining biochemical enrichment or selective labeling strategies with robust computational pipelines remains essential for accurate single-nucleotide resolution mapping of inosine. From a bioinformatic perspective, inosine sites are primarily inferred from A-to-G mismatches detected in RNA-seq data generated on Illumina platforms, as inosine is interpreted as guanosine during RT. Several dedicated computational tools have been developed to detect RNA editing events, including REDItools v1.3 [44], RES-Scanner [45], and SPRINT [46], as well as more general-purpose tools such as JACUSA [47]. All these approaches implement statistical frameworks to distinguish true A-to-I editing events from sequencing noise. These approaches typically rely on stringent filtering criteria, including base quality, read depth, strand bias, and mismatch frequency, and often require matched genomic DNA sequencing data to exclude SNPs and somatic variants. Additional challenges arise from misalignment in repetitive regions, particularly within Alu elements, which are highly enriched in editing sites. To address these issues, more advanced pipelines incorporate improved mapping strategies and annotation-aware filtering, as well as curated RNA editing databases such as DARNED, RADAR, and REDIportal v3 [48,49,50], which provide comprehensive catalogs of previously identified A-to-I editing sites for annotation and validation purposes. Despite these developments, the reliable detection of low-frequency editing events remains difficult, especially in lowly expressed transcripts, and continues to depend on the integration of multiple filtering steps and high sequencing depth.

### 2.2. Full-Length RNA Molecules and Transcript Isoforms

**Figure 3:**
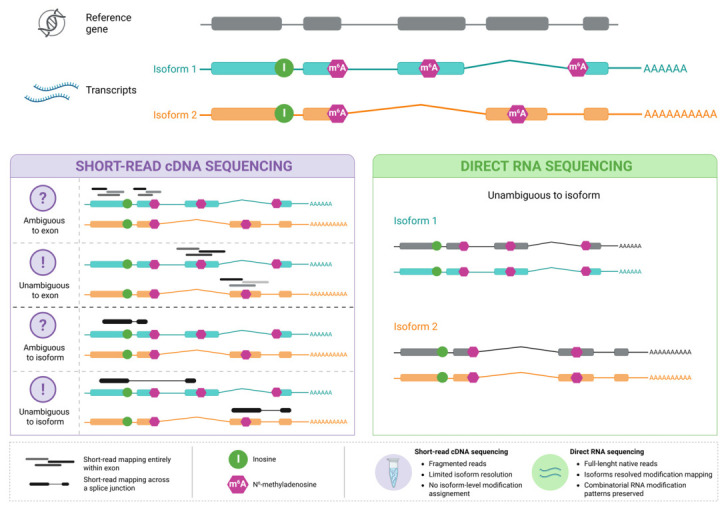
Hierarchical organization of epitranscriptomic information across RNA molecules. Schematic representation of how epitranscriptomic information is distributed across different levels of RNA organization, from individual nucleotides to full-length transcript isoforms. Alternative splicing generates multiple transcript isoforms from the same gene, each potentially carrying distinct modification patterns (e.g., m^6^A sites). Short-read cDNA sequencing provides high coverage for a specific modification, but often yields reads that are ambiguous for exon or isoform assignment, limiting the ability to resolve modification patterns at the transcript level. In contrast, direct RNA sequencing enables full-length, isoform-resolved analysis, allowing unambiguous mapping of modifications within specific transcript contexts. This highlights the importance of long-read approaches for capturing the complexity and combinatorial nature of epitranscriptomic regulation. Created in BioRender. Gallo, A. (2026) https://BioRender.com/w0gpxql, accessed on 8 June 2026.

Full-length RNA molecules and transcript isoforms provide an additional layer of resolution for studying RNA modifications beyond single-nucleotide analyses. While short-read RNA sequencing enables detection of modifications at single-nucleotide and exon resolution, it is inherently limited by RNA fragmentation, which complicates accurate isoform assignment and prevents direct reconstruction of co-occurring modification patterns within the same transcript molecule [51] (Figure 3). In contrast, long-read sequencing approaches, including direct RNA sequencing, preserve full-length transcript structure, enabling unambiguous isoform identification and the simultaneous detection of multiple RNA modifications along individual RNA molecules [32,51,52,53]. At this level of resolution, it becomes possible to move beyond site-centric analyses and investigate the spatial organization of modifications within transcripts, including their co-occurrence, relative distances, and distribution across functional domains. Single-molecule analyses further reveal epitranscriptomic heterogeneity by capturing combinations of modifications within individual RNA molecules, thereby defining distinct molecular states within transcript populations [54]. This is particularly relevant for both m^6^A and A-to-I editing, where modification patterns are often non-random and context-dependent. Region-based analyses complement this perspective by describing the enrichment of modifications across transcript features such as coding sequences, untranslated regions, introns, and repetitive elements. A-to-I editing, for example, is strongly enriched in double-stranded RNA structures formed by inverted Alu repeats, and global editing levels in these regions can be quantified using metrics such as the Alu editing index [55,56,57,58]. Similarly, m^6^A shows characteristic enrichment near stop codons and within 3′ untranslated regions [34,35]. At the sequence level, m^6^A deposition is enriched within the DRACH consensus motif [25,36], although only a subset of motifs is methylated, highlighting the importance of additional determinants such as RNA secondary structure and RNA-binding proteins. The advent of new-generation sequencing technologies, particularly nanopore-based platforms, is expected to open a wide range of possibilities for RNA “reading”. These include not only single-nucleotide resolution detection, but also the ability to link specific modifications to defined RNA isoforms (including also still unknown isoforms) and to identify combinatorial signature patterns involving multiple RNA modifications, either of the same type or distinct. Such advances will, in turn, drive the development of a broad spectrum of dedicated bioinformatic approaches that are still at an early stage of development, some of which will be discussed in the following paragraphs. RNA modifications are dynamic and context-dependent, varying across developmental stages, environmental conditions, and disease states [1,25]. Capturing this complexity requires analytical frameworks that integrate single-nucleotide resolution with isoform-level and single-molecule perspectives, enabling the study of modification co-occurrence, spatial organization, and combinatorial regulation within individual transcripts.

## 3. Illumina-Based Approaches for Epitranscriptomic Profiling

A wide range of computational strategies has been developed to investigate RNA modifications from Illumina sequencing data, reflecting both the diversity of biochemical assays and the intrinsic limitations of short-read technologies. These approaches differ substantially in their underlying assumptions, input data requirements, and resolution, and can be broadly categorized based on the type of signal used for modification inference.

### 3.1. Computational Frameworks for A-to-I RNA Editing Detection

For A-to-I RNA editing, most pipelines rely on the identification of A-to-G mismatches in RNA-seq data, followed by extensive filtering to distinguish true editing events from sequencing errors, mapping artifacts, and genomic polymorphisms. A key distinction among available tools lies in how they address this disambiguation step. Some methods, such as REDItools v1.3 [44], RES-Scanner2 [45], JACUSA [47], and RESIC [59], support or explicitly require matched DNA–RNA datasets, enabling direct comparison between genomic and transcriptomic sequences to exclude SNP-derived mismatches. These approaches typically incorporate multiple layers of filtering, including base quality, strand specificity, and coverage thresholds, and are considered among the most reliable for high-confidence editing detection. Other tools, including SPRINT v0.1.8 [46] and GIREMI [60], have been developed to operate without matched DNA data, relying instead on statistical modeling, clustering of mismatch patterns, or mutual information between editing sites to distinguish editing from genomic variation. While these approaches increase applicability in datasets lacking DNA sequencing, they may introduce higher false-positive rates or depend more heavily on model assumptions. Additional frameworks integrate external resources, such as SNP databases or curated RNA editing repositories (e.g., REDIportal v3 [50]), to further refine candidate sites. Overall, despite methodological differences, most pipelines converge on a common workflow involving mismatch detection, stringent filtering, and site-level quantification of editing frequencies.

#### REDItools-Based Workflows for A-to-I RNA Editing Detection-Using Matched DNA and RNA Sequencing

**Figure 4:**
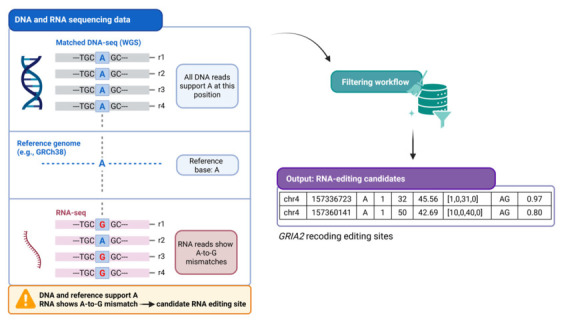
Workflow for RNA-DNA mismatch detection. Pre-aligned WGS and RNA-seq reads are compared to identify nucleotide mismatches, such as A-to-G changes consistent with A-to-I RNA editing. Candidate sites are filtered based on quality metrics and summarized in a table reporting genomic position, reference and alternative alleles, and editing levels. Created in BioRender. Gallo, A. (2026) https://BioRender.com/z5coli0, accessed on 8 June 2026.

In contrast to m^6^A detection, A-to-I RNA editing can be inferred directly from standard Illumina RNA-seq data by exploiting its characteristic sequencing signature (A-to-G mismatches) (Figure 4).

However, this signal is not specific to RNA editing, as identical mismatches can arise from genomic A/G polymorphisms (SNPs). For this reason, the most robust computational strategy relies on the comparison of RNA-seq and matched DNA-seq data derived from the same biological sample, allowing the exclusion of genomic variants and the identification of bona fide editing events.

In a typical REDItools-based workflow [61], the analysis proceeds through the following steps:

1. Independent alignment of RNA-seq and DNA-seq reads to the reference genome using splice-aware (STAR for RNA) and standard (BWA-MEM for DNA) aligners
2. Identification of candidate mismatches in RNA-seq data, with a focus on A-to-G substitutions
3. Filtering against matched DNA data, removing positions where the corresponding genomic locus shows an A/G polymorphism
4. Application of stringent quality filters, including:
  - minimum read coverage
  - base quality thresholds
  - strand bias correction
  - mapping quality filters
5. Annotation and post-processing, including comparison with known RNA editing databases (e.g., REDIportal) and genomic context filtering

These steps are implemented within REDItools v1.3, which provides a flexible framework for parsing aligned reads and computing nucleotide frequencies at each genomic position, enabling quantitative estimation of editing levels. The integration of matched DNA data represents a critical requirement for high-confidence editing detection, as it allows the systematic removal of false positives arising from germline or somatic variants. In the absence of matched DNA, alternative strategies rely on population variant databases and additional filtering criteria, but these approaches are inherently less specific.

Overall, to provide a clearer conceptual framework for A-to-I RNA editing analysis, current computational approaches can be broadly classified into four main categories: (i) methods for the site-level detection of editing events, (ii) tools for differential editing analysis across biological conditions, (iii) database-supported annotation and filtering pipelines, and (iv) strategies integrating matched DNA/RNA sequencing data. These analytical frameworks address different but interconnected challenges associated with accurately identifying *bona fide* editing events. In particular, a major issue in A-to-I editing analysis is the discrimination of true editing sites from potential confounding factors, including genomic variants, sequencing errors, mapping artifacts, and transcriptome/genome alignment biases. While site-detection tools primarily focus on sensitivity and positional accuracy, annotation-based and matched DNA/RNA approaches are especially important for reducing false positives and improving confidence in editing calls.

### 3.2. Computational Strategies for m^6^A Detection from Illumina Data

In contrast to RNA editing, m^6^A does not generate a universal and direct sequencing signature in standard Illumina data; therefore, its detection relies on indirect signals or experimentally induced modifications, and computational approaches are tightly coupled to the underlying experimental protocol. For antibody-based methods such as MeRIP-seq or m^6^A-seq, bioinformatic analysis is primarily based on peak-calling and enrichment strategies, where regions with increased read coverage in immunoprecipitated samples relative to input controls are identified as putatively methylated. Common tools for this purpose include exomePeak [62] and MACS2 [63], which provide transcriptome-wide maps of m^6^A distribution at a typical resolution of ~100–200 nucleotides but with limited site-level precision and quantitative accuracy. To overcome these limitations, antibody-independent or enhanced-resolution strategies have been developed, each requiring dedicated computational pipelines. Enzyme-based methods such as MAZTER-seq and m^6^A-REF-seq exploit sequence-specific cleavage patterns to infer methylation status, whereas fusion-based approaches such as DART-seq rely on enzyme recruitment to induce mutation signatures proximal to modified nucleotides. In parallel, chemical conversion strategies exploit differential reactivity between modified and unmodified adenosines to generate mutation-based readouts; among these, GLORI [39] represents a robust implementation that enables single-nucleotide resolution and quantitative m^6^A profiling through glyoxal protection of guanosines and nitrite-mediated deamination of unmodified adenosines. Crosslinking-based approaches such as miCLIP further enable near single-nucleotide resolution by introducing truncation or mutation signatures at modification sites [36]. Regardless of the experimental strategy, downstream analyses commonly integrate sequence context (notably DRACH motifs), transcript positional information (e.g., enrichment near stop codons and within 3′ UTRs), and statistical modeling to improve confidence in site identification [34]. More generally, chemical conversion-based approaches such as GLORI have introduced a conceptual shift by transforming m^6^A detection into a direct base-resolution readout analogous to bisulfite sequencing in DNA methylation analysis. Given the heterogeneity of available strategies, the choice of computational framework depends on the experimental design, biological question, and desired resolution. In this context, representative analytical pipelines include GLORI-based workflows for quantitative base-resolution m^6^A profiling, highlighting the shift from enrichment-based approaches toward direct, mutation-based, and quantitative detection strategies.

#### GLORI-Seq for Absolute Quantification of m^6^A at Single-Base Resolution

**Figure 5:**
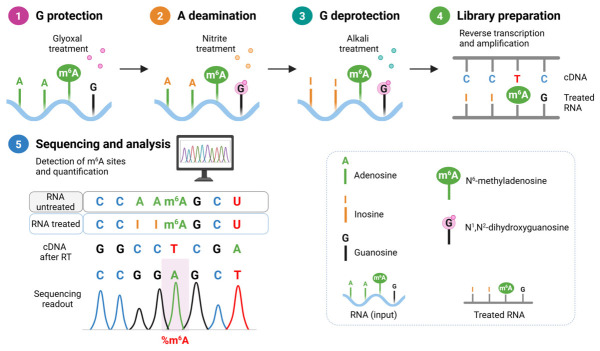
Workflow for the identification of m^6^A using GLORI-seq. Schematic representation of the GLORI-seq approach, in which glyoxal protects guanosines and subsequent nitrite-mediated deamination converts unmodified adenosines to inosines, while m^6^A residues are resistant to conversion. Following RT and sequencing, inosines are read as guanosines, enabling the identification of m^6^A sites as positions retaining an adenosine signal (A) in the converted transcriptome. Quantification is based on the relative A-to-G conversion rates at each site. Created in BioRender. Gallo, A. (2026) https://BioRender.com/e2rphre, accessed on 8 June 2026.

GLORI is a sequencing-based method that enables transcriptome-wide, single-nucleotide resolution mapping and absolute quantification of m^6^A, conceptually analogous to bisulfite sequencing for DNA methylation. The method is based on a selective chemical deamination reaction in which unmodified adenosines are converted to inosines, while m^6^A residues remain chemically protected and unmodified (Figure 5). This is achieved through a glyoxal- and nitrite-mediated reaction that combines two key steps: (i) protection of guanosines through glyoxal adduct formation, and (ii) efficient deamination of adenosines under optimized conditions, resulting in near-complete A-to-I conversion (~98–99%) without affecting m^6^A. Following chemical treatment, RNA is reverse transcribed and sequenced using standard Illumina workflows. During RT, inosine is read as guanosine, leading to A-to-G conversions at positions corresponding to unmethylated adenosines, whereas m^6^A residues are retained as A. As a result, m^6^A sites can be directly identified as positions sequenced as adenosines after the conversion treatment.

At the computational level, GLORI relies on a dedicated pipeline that performs:

1. Preprocessing and deduplication of reads, often leveraging unique molecular identifiers (UMIs) to control for PCR bias
2. Alignment to a converted (ternary) reference genome, designed to accommodate systematic A-to-G changes
3. Site-level inference of m^6^A, based on the proportion of reads retaining A at each position
4. Quantification of methylation stoichiometry, calculated as the fraction of A over total coverage at each site

This framework enables not only precise localization of m^6^A sites but also absolute quantification of modification levels, overcoming the major limitation of antibody-based methods that provide only relative enrichment signals. Importantly, GLORI has been shown to achieve high accuracy across sequence contexts and to detect hundreds of thousands of m^6^A sites with high reproducibility. From a practical perspective, the protocol involves RNA fragmentation, chemical treatment, library preparation using modified eCLIP-like workflows, and downstream analysis using the GLORI-tools pipeline.

## 4. Long-Read Approaches for Epitranscriptomic Profiling

### 4.1. Long-Read RNA Sequencing: Platform-Specific Opportunities and Limitations

Long-read sequencing technologies provide important opportunities for transcriptomic and epitranscriptomic studies, but their contributions are fundamentally different since they depend strongly on the molecule being sequenced and on the type of signal that is measured. In this context, it is important to distinguish cDNA-based long-read sequencing from native RNA sequencing. PacBio SMRT sequencing detects fluorescent signals generated during nucleotide incorporation by a DNA polymerase, enabling long-read sequencing at the single-molecule level [64]. In standard transcriptomic applications, including Iso-Seq [65] and Kinnex workflows, RNA molecules are first reverse-transcribed into full-length cDNA, which is then sequenced using the PacBio platform. In these workflows, the sequencing template is cDNA, rather than native RNA, and standard transcriptomic protocols do not preserve RNA base chemistry in the final sequencing molecule. PacBio HiFi sequencing provides highly accurate consensus reads through circular consensus sequencing of SMRTbell templates, in which the same molecule is read multiple times to generate a high-confidence sequence [66]. These approaches are particularly powerful for transcript isoform discovery, isoform quantification, fusion transcript detection, allele-specific expression, and transcriptome annotation.

From an epitranscriptomic perspective, the usefulness of cDNA-based long-read sequencing depends on the type of RNA modification being investigated. For A-to-I RNA editing, PacBio Iso-Seq/Kinnex data can still be informative because inosine is read as guanosine during RT, resulting in A-to-G mismatches in the sequenced cDNA. The long-read format may improve mapping confidence, particularly in repetitive or isoform-complex regions, and may help assign editing events to specific transcript isoforms. In addition, single-molecule long reads can support the analysis of multiple editing sites occurring within the same transcript molecule or isoform. However, this logic does not apply equally to non-mutagenic RNA modifications such as m^6^A. Because standard PacBio Iso-Seq/Kinnex workflows sequence cDNA rather than native RNA, native RNA base modifications are generally not directly preserved as chemical features in the final sequencing molecule.

Nevertheless, PacBio-based strategies have also been explored for RNA modification analysis. In particular, single-molecule real-time detection of reverse transcription (SMRT-RT) uses reverse transcriptase rather than the DNA polymerase used in conventional SMRT sequencing, enabling real-time monitoring of RNA-to-cDNA synthesis [67]. In this setting, RNA modifications can alter reverse-transcription kinetics, for example by inducing changes in incorporation dynamics or enzyme pausing. This principle has been used to investigate modification-sensitive signatures, including those associated with m^6^A. However, SMRT-RT remains largely a specialized or proof-of-concept approach and has not become a routine transcriptome-wide workflow for m^6^A detection.

By contrast, Oxford Nanopore Technologies (ONT) DRS interrogates native RNA molecules without RT or PCR amplification, thereby retaining RNA base chemistry during sequencing [32]. This distinction makes the ONT DRS conceptually closer to direct epitranscriptomic profiling than cDNA-based long-read approaches. The following section therefore focuses on how native RNA molecules are processed by nanopore sequencing and how modification-associated signals can be extracted computationally.

### 4.2. Nanopore-Based Approaches for Epitranscriptomic Profiling

Nanopore DRS is a single-molecule approach that sequences native RNA strands without RT or PCR amplification, thereby preserving RNA base chemistry and enabling the joint analysis of transcript identity, abundance, and nucleotide modifications from the same dataset [32,68]. In the standard DRS workflow, polyadenylated RNA is captured via the 3′ poly(A) tail and threaded through a biological nanopore by sequencing adapters and a motor enzyme that regulates translocation, so that the RNA strand is read in the 3′ → 5′ direction [32,68].

As the RNA molecule passes through the nanopore under an applied voltage, it partially blocks ion flow and generates a time-resolved electrical current trace, commonly referred to as a “squiggle”. Importantly, because the pore accommodates more than one nucleotide at a time, the measured current reflects the combined physicochemical contribution of a k-mer within the sensing region rather than that of a single base [68,69,70]. This k-mer dependence is a central feature of nanopore epitranscriptomics, as the same chemical modification may produce different signal perturbations depending on the local sequence context. In addition, apparent signal shifts may arise from RNA secondary structure, translocation irregularities, and run-to-run variation in pore behavior, all of which complicate direct interpretation of the raw signal [67,69,70,71]. Unlike fluorescence-based sequencing, nanopore DRS measures a continuous analog signal whose interpretation depends on both local sequence composition and motor kinetics [68,69]. The motor protein advances the RNA in discrete steps that are not perfectly uniform, leading to variability in dwell time and event segmentation [69,72]. Modifications may influence not only the mean current level for a local k-mer but also the distributional properties of the signal, and, in some contexts, the translocation kinetics. However, these same properties can be influenced by non-modification factors (e.g., structured regions, homopolymers, RNA damage, pore-to-pore variability), making robust inference inherently statistical and control-dependent [72,73,74].

The accuracy and interpretability of RNA modification detection using nanopore direct RNA sequencing are strongly influenced by several interconnected methodological factors. In particular, modification calls depend on the specific sequencing chemistry employed, the version and architecture of the basecalling software, and the underlying trained models used for signal interpretation [75,76]. In addition, the choice of probability thresholds applied during post-processing can substantially affect the balance between sensitivity and specificity of detected modification sites [54].

A critical aspect of nanopore-based modification calling is its reliance on model-derived outputs, often represented as MM/ML tags, which encode per-read probabilities of modified bases inferred from deviations in the raw electrical signal. However, these probabilities are inherently model-dependent and reflect the training data and assumptions embedded in the basecaller. As a consequence, differences in model versions or training strategies can lead to variability in modification detection performance across datasets [76,77].

Furthermore, nanopore sequencing provides single-molecule resolution, which enables the detection of heterogeneous modification patterns at the read level, but also introduces additional complexity in interpretation due to intrinsic signal noise and stochastic variation. Importantly, current models support only a subset of RNA modifications for which adequate training and validation data are available, limiting the generalizability of predictions to uncharacterized or less well-studied modifications.

Overall, these considerations highlight that nanopore-based RNA modification detection remains strongly model-dependent, and its performance is contingent on the availability of robust training datasets, well-calibrated models, and appropriate validation strategies.

### 4.3. Computational Frameworks for Modifications Detection

Standard analysis of Oxford Nanopore Technologies (ONT) direct RNA sequencing data begins with pod5 files, which encapsulate raw signal-level data generated during the sequencing process. The initial step involves basecalling, a computational process that translates the observed ionic current traces from the nanopore into nucleotide sequences using a neural-network model, typically using tools like Guppy v2.0.0, Bonito v1.1.0 or Dorado v2.0.0 for high-accuracy outputs [68,73,78]. This is followed by alignment of the basecalled reads to a reference transcriptome or genome, often with minimap2 or similar graph-based mappers optimized for long-read data [68]. The final step consists of the quantification of RNA modifications [69,73]. Computational methods for modification detection from DRS data can be grouped into three main paradigms. (1) Mismatch/basecalling-error approaches exploit systematic patterns of miscalls (mismatches/indels) associated with specific modifications, using features extracted from basecalled alignments as proxies for modification signal; representative frameworks include EpiNano and general-purpose statistical toolkits such as JACUSA2 v2.0.4, which support modification-oriented analyses across sequencing assays and can operate on long-read data [73,79,80,81]. (2) Signal-level modeling approaches leverage raw current features (e.g., current level distributions, dwell/kinetics proxies, and trace shape) mapped to reference coordinates to build statistical or machine-learning models that detect modification-associated deviations even when canonical basecalling remains accurate; classic and widely used pipelines include Tombo (signal anchoring and testing/modeling at candidate sites) and comparative signal-feature frameworks such as Nanocompore and related approaches that identify modified positions by contrasting signal feature distributions across conditions (e.g., WT *versus* writer/eraser perturbations), thereby helping distinguish true modification signatures from run- or pore-specific noise [54,73,74,82]. (3) Modification-aware basecalling integrates modification inference directly into the basecalling stage via models trained on labeled signal data, outputting per-read modification probabilities during sequence decoding rather than requiring a separate *post hoc* caller; recent work shows that basecaller choice and training strategy can materially influence both mapping performance and modification detectability, reinforcing the notion that the basecaller is part of the “measurement model” for epitranscriptomic inference [76,78,83]. Importantly, in current standard practice the primary sequence in the BAM typically remains in the canonical alphabet, while modified-base annotations are stored as optional tags in SAM/BAM files that encode modified-base positions and per-base probabilities/likelihoods, enabling downstream quantification of modifications occupancy at sites and comparison between genome- *vs.* transcriptome-aligned analyses.

### 4.4. Modification Detection Using ONT-Developed Tools

Oxford Nanopore Technologies provides an integrated software ecosystem for DRS, including Dorado for basecalling (with optional modified-base models) and Modkit v0.6.4 for downstream summarization and quantification of modified-base calls [84,85].

**Figure 6:**
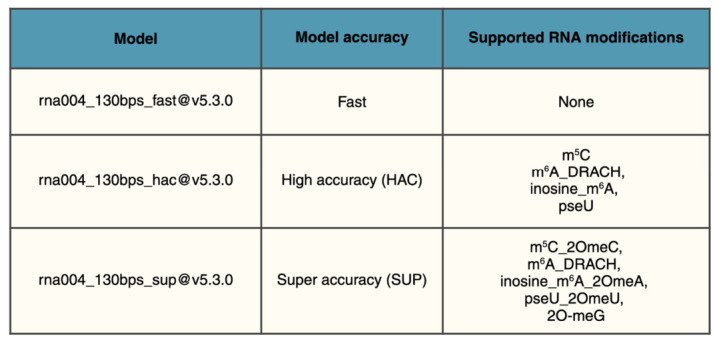
Overview of ONT Dorado RNA basecalling models and their supported RNA modifications. Summary of ONT RNA004 v5.3.0 (latest release) basecalling models, their accuracy profiles, and supported RNA modification calls. While the fast model is optimized for speed and does not include modification detection, the hac and sup models enable increasingly comprehensive modification-aware basecalling. In particular, the sup model combines the highest basecalling accuracy with the widest range of supported RNA modifications, although this improvement comes at a higher computational cost. Created in BioRender. Gallo, A. (2026) https://BioRender.com/2rn4dqd, accessed on 21 April 2026.

In a commonly adopted modification-aware workflow, basecalling is performed with Dorado basecaller using a high-accuracy RNA model together with a modified-based model configured to call multiple marks in the same run (e.g., rna004_130bps_sup@v5.1.0_inosine_m^6^A@v1 for m^6^A and inosine) (Figure 6). The resulting reads are aligned with Dorado aligner to either a genome reference (with a splice aware preset) or a transcriptome reference, followed by sorting and indexing. To mitigate artifactual calls driven by ambiguous or low-confidence mappings, pipelines commonly restrict analysis to primary mapped reads and apply explicit mapping- and base/read-quality filters prior to quantification [78]. Importantly, modification-aware basecalling typically retains canonical letters in the primary sequence and encodes modified-base calls using standardized SAM/BAM optional tags most commonly MM (modified-base coordinates) and ML (per-base probabilities/likelihoods) as defined by the SAM/HTS specifications, enabling downstream quantification directly from alignment files [85]. Site-level quantification can be performed with tools such as Modkit, which aggregate per-read modification probabilities into per-position estimates (pileup-style summaries) for each modification type, optionally using modification-specific probability thresholds and *post hoc* filters on coverage and minimum supporting observations to stabilize occupancy estimates. Because modified-base predictions are stored per read, downstream analyses can move beyond pileup-style aggregation to quantify within-molecule co-occurrence of multiple modified sites and assess molecule-to-molecule heterogeneity [54,78,86]. Running parallel quantification on genome- and transcriptome-aligned BAMs provides a practical way to evaluate how reference choice impacts site assignment in isoform-complex regions, where long reads offer a key advantage over short-read approaches.

### 4.5. Multi-Modification Detection Strategies

Emerging sequencing and computational approaches are increasingly aimed at the simultaneous detection of multiple RNA modifications within the same transcript. In this context, third-generation sequencing technologies, particularly Oxford Nanopore DRS, have rapidly gained prominence due to their unique ability to read native RNA molecules without prior RT or amplification, thereby preserving the original epitranscriptomic information [69]. Nanopore-based approaches enable the direct detection of RNA modifications by measuring characteristic alterations in ionic current signals as individual RNA molecules translocate through the pore. This has the potential to enable the interrogation of multiple co-occurring modifications, such as m^6^A, pseudouridine (Ψ), 5-methylcytosine (m^5^C), and A-to-I editing, at single-molecule resolution within the same transcript, providing an unprecedented view of epitranscriptomic heterogeneity [54]. Importantly, recent methodological developments and basecalling frameworks have demonstrated the capability of simultaneously identifying several distinct modifications on individual RNA strands, highlighting the feasibility of integrated, multi-mark detection strategies [54,87]. Parallel advances in computational biology, particularly machine learning and deep learning-based signal decoding, have further enhanced the sensitivity and specificity of nanopore-derived modification calls. These models leverage raw signal features and systematic basecalling errors to distinguish chemically diverse modifications and to perform *ab initio* multi-modification detection within a unified analytical framework [88]. The ability to profile multiple RNA modifications simultaneously is particularly relevant for understanding epitranscriptomic crosstalk, as increasing evidence suggests that distinct modifications can co-occur on the same RNA molecule and function cooperatively or antagonistically to regulate RNA metabolism, stability, translation, and splicing [89]. Such combinatorial patterns are emerging as a key layer of post-transcriptional gene regulation that cannot be captured by single-mark profiling approaches. Despite these advances, accurate discrimination among chemically similar modifications and robust quantitative estimation remain significant challenges. Signal overlap, context dependency, and variability across sequencing chemistries still require substantial methodological refinement, as well as rigorous validation using orthogonal techniques such as mass spectrometry. Consequently, continued efforts in standardization, benchmarking, and integration of multi-omics datasets will be essential to fully decode the complexity of transcript-specific epitranscriptomic landscapes.

### 4.6. Critical Comparison of Methodological Approaches

Illumina-based RNA sequencing remains the gold standard for transcriptome-wide RNA editing quantification due to its high accuracy, depth, and mature computational ecosystem. This approach is particularly advantageous for detecting low-frequency editing events and performing differential editing analyses across large cohorts [90]. However, the short-read nature of Illumina sequencing limits the accurate reconstruction of full-length transcript isoforms and complicates the analysis of repetitive genomic regions, including Alu-rich loci frequently associated with A-to-I editing. This approach misses the full transcript and identifies one mark at a time (such as Inosine or m^6^A). In contrast, long-read sequencing technologies such as Oxford Nanopore Technologies (ONT) and PacBio Iso-Seq enable the characterization of full-length transcripts and easier identification of isoform-specific RNA editing patterns. ONT additionally offers the possibility of direct RNA sequencing, avoiding RT and PCR amplification biases and identifying multiple marks [91,92]. Nevertheless, higher raw error rates, particularly in ONT datasets, still represent a major limitation for accurate editing detection and require dedicated computational correction and validation strategies. Of note, PacBio HiFi sequencing provides improved base accuracy compared with ONT and allows reliable full-length transcript reconstruction, although at the expense of lower throughput and increased sequencing costs. Consequently, PacBio approaches are particularly suitable for high-confidence transcript isoform analyses, whereas ONT platforms are often preferred for exploratory analyses of transcript complexity and particularly for RNA modifications [93]. Overall, the choice of sequencing platform should be guided by the biological question and experimental design. Illumina sequencing is currently preferable for large-scale quantitative analyses of specific RNA marks and for studies requiring high sensitivity and statistical power. Conversely, long-read approaches are particularly advantageous when transcript isoform diversity, editing co-occurrence within single molecules, or complex transcript analysis are central to the study objectives (Table 1). Hybrid strategies combining short- and long-read sequencing may provide the most comprehensive framework for accurate and biologically informative RNA editing analyses.

### 4.7. Limitations and Validated Applications

Illumina-based methods have been extensively validated for transcriptome-wide identification of A-to-I editing events in cancer, neurological disorders, and immune-related diseases, owing to their high sequencing depth, low per-base error rates, and mature analytical pipelines [94,95]. These approaches are particularly suitable for large-scale quantitative analyses and for detecting low-frequency editing events. However, false positives may arise from mapping artifacts, SNP contamination, or RT errors, thus requiring stringent filtering procedures and, whenever possible, matched genomic DNA controls. Long-read sequencing approaches are particularly useful for transcript-resolved analyses, including isoform-specific editing, alternative splicing-associated editing events, and editing within repetitive regions. By preserving full-length RNA information, these technologies enable more accurate reconstruction of transcript complexity. Current limitations include higher sequencing costs, lower throughput, and higher intrinsic sequencing error rates compared with short-read platforms. Overall, short-read sequencing is generally preferred for accurate quantification and large cohort studies, whereas long-read sequencing is better suited for transcript-level characterization and complex transcriptomic architectures.

### 4.8. Validation and Benchmarking

Robust validation and benchmarking are essential to ensure the reliability of RNA modification detection approaches. A key strategy involves the use of *in vitro* transcribed (IVT) RNA controls, which provide unmodified reference sequences for estimating false positive rates and assessing baseline signal noise [83]. Complementary to this, knockout or knockdown models targeting specific writer or eraser enzymes enable the evaluation of signal specificity by testing whether the expected modification signal is lost upon perturbation, while IVT-derived transcripts can serve as additional negative controls in comparative frameworks [54]. Synthetic spike-in standards containing known modification sites further support quantitative benchmarking and enable calibration of detection performance across experiments, facilitating systematic performance assessment across modification types and sequence contexts. In addition to internal controls, orthogonal validation approaches are widely used to confirm computationally inferred modification sites using independent experimental techniques, such as mass spectrometry, which is considered a gold standard for the accurate detection and quantification of modified ribonucleosides [96]. Finally, cross-platform comparisons between short-read and long-read sequencing technologies provide an additional layer of validation, allowing assessment of consistency and robustness of detected signals across different experimental and analytical frameworks, while also highlighting platform-specific biases and limitations [97].

## 5. Open Bioinformatic Challenges and Recommendations

Despite significant progress, several challenges remain in the analysis of RNA modifications. A major limitation is the lack of comprehensive ground-truth datasets, which complicates the benchmarking and validation of computational methods. Performance estimates are often influenced by dataset composition and evaluation criteria, underscoring the need for standardized benchmarks. To address this limitation, the field would benefit from the coordinated development of community-curated reference datasets that include synthetic RNA controls, orthogonally validated modification sites, and standardized experimental protocols for data generation. In this context, a large-scale international initiative such as the Human RNome Project has recently been launched with the explicit goal of defining the full repertoire of RNA molecules and their chemical modifications in the human transcriptome, while establishing reference systems, shared standards, and benchmarking frameworks for cross-platform comparison [98]. The consortium is actively developing coordinated benchmarking efforts based on common reference samples and multi-technology profiling strategies, enabling reproducible evaluation of RNA modification detection methods across sequencing platforms. These community-driven resources are expected to provide standardized datasets and interoperable formats, thereby enabling consistent cross-study benchmarking, reducing dataset-specific biases, and accelerating the development of robust and generalizable analytical pipelines [98].

For A-to-I editing, distinguishing true editing events from sequencing errors, genomic variants, and mapping artifacts remains challenging and requires rigorous filtering strategies. Future methodological development should prioritize the integration of multi-layered filtering frameworks combining genomic variant calling, stranded transcript-aware alignment, and site-level statistical confidence scoring to improve specificity in editing detection [74,99].

For m^6^A and other modifications, accurate estimation of stoichiometry remains difficult, as these marks are often present at sub-stoichiometric levels [54]. Addressing this challenge will require the development of quantitative calibration standards, improved probabilistic modeling of signal distributions, and the systematic use of spike-in controls to enable absolute or semi-quantitative stoichiometry estimation.

In addition, discrepancies between sequencing platforms pose challenges for data integration, as each technology captures different aspects of the epitranscriptome [77,97].

A key priority moving forward is the establishment of harmonization frameworks that enable cross-platform normalization and joint analysis, potentially through shared feature representations or transfer learning-based approaches.

These issues are particularly evident in Nanopore-based analyses, where model dependence, sequence-context effects, and variability across experimental conditions further complicate interpretation. ONT has released successive direct RNA sequencing chemistries (RNA001/RNA002/RNA004), which have improved throughput and read accuracy but have also required re-evaluation and recalibration of modification-calling performance across datasets generated with different chemistries [76,87,100]. This highlights the need for version-aware benchmarking frameworks that explicitly account for chemistry-specific biases and ensure comparability across sequencing generations.

Model training remains a key bottleneck, as modification-aware basecallers are typically trained on paired unmodified/modified references or wt/perturbation-based designs (e.g., writer KO), while training data and calibration procedures are not always disclosed in sufficient detail to enable full independent reproduction [101]. To improve transparency and reproducibility, future tool development should adopt standardized reporting of training datasets, model architectures, and calibration strategies, ideally following community-agreed guidelines similar to those used in other omics fields. In addition, publicly available benchmark training sets should be established to reduce dependence on proprietary or incompletely described datasets.

In practice, modification probability thresholds and IVT controls are frequently required to control false-positive rates, and benchmarking studies continue to highlight substantial variability across callers and experimental conditions [87,101]. Taken together, these limitations show that progress in the field depends not only on improved sequencing performance, but also on greater methodological transparency, reproducibility, and standardization. Collectively, these considerations suggest the need for an integrated quality-control and benchmarking ecosystem that defines minimal reporting standards, recommended validation pipelines, and shared performance metrics across tools and platforms, an effort that is now being actively pursued by international initiatives such as the Human RNome Project, which aims to establish community-wide reference datasets and standardized frameworks for RNA modification analysis [98].

### Clinical Translation Considerations

The rapid evolution of RNA modification profiling technologies is progressively opening new opportunities for clinical applications, including biomarker discovery, patient stratification, and therapeutic target identification. In this context, RNA editing, particularly A-to-I editing mediated by ADAR enzymes, represents one of the most extensively characterized epitranscriptomic processes with direct clinical relevance. Dysregulation of ADAR activity has been implicated in multiple pathological conditions, including cancer, neurological disorders, and autoimmune or inflammatory diseases, where altered editing landscapes contribute to transcriptome instability and aberrant immune signaling. Large-scale profiling studies have suggested that RNA editing patterns may act as disease-specific molecular signatures. Integrative analyses combining RNA-seq and dedicated editing-detection pipelines have revealed widespread recoding events and editing-associated dysregulation across tumor types, supporting the use of editing profiles as potential biomarkers and prognostic indicators [50]. In parallel, neurological disorders such as epilepsy, ALS, and psychiatric diseases have been associated with impaired ADAR-mediated editing at key sites (e.g., GRIA2 Q/R site), highlighting the functional importance of editing fidelity in neuronal homeostasis [21]. More broadly, altered epitranscriptomic signatures, including m^6^A, m^5^C, pseudouridine, and A-to-I editing have been linked to diverse disease phenotypes, reinforcing the concept that combinatorial RNA modification patterns may provide complementary information to single-mark analyses. Recent studies leveraging direct RNA sequencing and integrative computational frameworks have further suggested that co-occurrence of multiple RNA modifications can influence transcript-specific regulation and may provide enhanced diagnostic and predictive power in clinical settings [54,89]. Despite these promising advances, several challenges still limit clinical implementation. These include substantial variability among experimental protocols, lack of standardized and reproducible computational pipelines for the detection of RNA editing and other epitranscriptomic marks, as well as persistent issues related to sequencing costs and cross-platform reproducibility. Additionally, accurate quantification of editing levels and discrimination between true biological events and technical artifacts remain critical bottlenecks, particularly in low-frequency editing contexts. Future improvements in sequencing accuracy, especially through direct RNA nanopore technologies and enhanced basecalling algorithms, together with robust analytical standardization and large-scale validation studies across patient cohorts, will be essential for translating RNA modification profiling into clinical practice. Ultimately, the integration of RNA editing and multi-modification epitranscriptomic data into precision medicine frameworks may provide a more comprehensive and dynamic view of disease biology, enabling more accurate stratification and novel therapeutic targeting strategies.

To facilitate clinical implementation, future efforts should prioritize the development of standardized experimental protocols and benchmarking datasets, the establishment of reproducible computational pipelines, and large-scale validation studies across independent patient cohorts. In parallel, the integration of epitranscriptomic information with other multi-omics data and clinically interpretable decision-support frameworks will be essential for translating RNA modification profiling into routine precision medicine applications.

## 6. Future Perspectives

Future advances in epitranscriptomics will depend on integrating complementary sequencing technologies, improving model generalization, and developing standardized benchmarking frameworks.

In particular, a major next step will be the implementation of unified analytical frameworks that combine short-read, long-read, and direct RNA sequencing data to improve both sensitivity and resolution of RNA modification detection. Bridging the gap between signal-level detection and biological interpretation will be essential to translate epitranscriptomic insights into biological and clinical applications. To achieve this, future research should prioritize the development of interpretable computational models that link raw signal features to mechanistic biological outcomes, enabling more robust functional annotation of RNA modifications. From a translational perspective, key actionable priorities include the validation of RNA modification signatures in independent cohorts, the establishment of clinically relevant assay standards, and the development of robust pipelines for biomarker discovery. In parallel, the field should move toward multi-modification and isoform-resolved analyses, enabling a more comprehensive view of epitranscriptomic regulation at single-transcript resolution. Overall, these developments outline a clear roadmap for the field, moving from method-centric innovation toward standardized, reproducible, and clinically deployable epitranscriptomic profiling strategies.

## 7. Conclusions

Epitranscriptomic analysis is inherently shaped by the sequencing technologies and computational strategies used to interrogate RNA modifications. Illumina and Nanopore approaches provide complementary perspectives, enabling population-level quantification and molecule-resolved analyses, respectively. Their integration will be essential to achieve a comprehensive understanding of RNA modification landscapes and their functional implications.

## Figures and Tables

**Table 1:** Comparison of sequencing technologies for RNA modification analysis. Key parameters—including read length, base accuracy, isoform resolution, detection of editing clusters, throughput, and cost—are summarized for Illumina, ONT, and PacBio Iso-Seq, alongside their principal strengths, limitations, and recommended applications.

Feature	Illumina Short-Read Sequencing	Oxford Nanopore Technologies (ONT)	PacBio Iso-Seq
Read length	50–300 bp	Up to several kb or full-length transcripts	Full-length transcripts
Base accuracy	Very high (>99%)	Moderate, improving with newer chemistries/basecalling	High (HiFi reads > 99%)
Isoform resolution	Limited	Excellent	Excellent
Detection of editing clusters	Partial/local	Comprehensive within single molecules	Comprehensive within single molecules
Throughput	Very high	Moderate–high	Moderate
Cost per sample	Relatively low	Moderate	High
Main strengths	Sensitive quantification and mature pipelines. Usually focused on a single modification class per experiment	Direct RNA sequencing and long-range transcript information. Potential simultaneous detection of multiple marks.	Accurate full-length transcript characterization
Main limitations	Poor reconstruction of full isoforms; mapping ambiguity in repetitive regions	Higher raw error rate	Lower throughput and higher sequencing cost
Recommended applications	Population-level editing quantification; differential editing analyses	Isoform-specific editing and direct RNA modification studies. Potentially multiple marks.	High-confidence full-length transcript and isoform analyses

## Data Availability

No new data were created or analyzed in this study. Data sharing is not applicable to this article.

## Abbreviations

The following abbreviations are used in this manuscript:

ADAR	Adenosine DeAminase Acting on RNA
ALS	Amyotrophic Lateral Sclerosis
A-to-I	Adenosine to Inosine
circRNA	Circular RNA
CLIP	Crosslinking and immunoprecipitation
DRACH	D=A/G/U, R=A/G, H=A/C/U consensus motif
DRS	Direct RNA sequencing
EndoV	Endonuclease V
IVT	In vitro transcription
lncRNA	Long non-coding RNA
m^5^C	5-methylcytosine
m^6^A	N6-methyladenosine
miRNA	MicroRNA
MeRIP-seq	Methylated RNA Immunoprecipitation sequencing
NGS	Next-Generation Sequencing
ONT	Oxford Nanopore Technologies
PacBio	Pacific Biosciences
PCR	Polymerase chain reaction
rRNA	Ribosomal RNA
RT	Reverse transcription
SBS	Sequencing by synthesis
SMRT	Single-molecule Real-time
snRNA	Small nuclear RNA
snoRNA	Small nucleolar RNA
SNP	Single-nucleotide polymorphism
UMI	Unique molecular identifier
UTR	Untranslated region
Ψ	Pseudouridine
